# Lifestyle physical activity among urban Palestinians and Israelis: a cross-sectional comparison in the Palestinian-Israeli Jerusalem risk factor study

**DOI:** 10.1186/1471-2458-12-90

**Published:** 2012-01-30

**Authors:** Dafna Merom, Ronit Sinnreich, Vartohi Aboudi, Jeremy D Kark, Hisham Nassar

**Affiliations:** 1 School of Science and Health, University of Western Sydney, Locked Bag 1797, Penrith NSW 2571, Australia; 2 Epidemiology Unit, Hebrew University-Hadassah School of Public Health and Community Medicine, Jerusalem, Israel; 3 St Joseph Hospital, East Jerusalem, Jerusalem, Israel; 4 Cardiology Department, Hadassah-Hebrew University Medical Center and Cardiology Consultant, St Joseph Hospital, East Jerusalem, Jerusalem, Israel; 5 IVF Department, Hadassah-Hebrew University Medical Center, Jerusalem, Israel

## Abstract

**Background:**

Urban Palestinians have a high incidence of coronary heart disease, and alarming prevalences of obesity (particularly among women) and diabetes. An active lifestyle can help prevent these conditions. Little is known about the physical activity (PA) behavior of Palestinians. This study aimed to determine the prevalence of insufficient PA and its socio-demographic correlates among urban Palestinians in comparison with Israelis.

**Methods:**

An age-sex stratified random sample of Palestinians and Israelis aged 25-74 years living in east and west Jerusalem was drawn from the Israel National Population Registry: 970 Palestinians and 712 Israelis participated. PA in a typical week was assessed by the Multi-Ethnic Study of Atherosclerosis (MESA) questionnaire. Energy expenditure (EE), calculated in metabolic equivalents (METs), was compared between groups for moderate to vigorous-intensity physical activity (MVPA), using the Wilcoxon rank-sum test, and for domain-specific prevalence rates of meeting public health guidelines and all-domain insufficient PA. Correlates of insufficient PA were assessed by multivariable logistic modeling.

**Results:**

Palestinian men had the highest median of MVPA (4740 METs-min_*_wk^-1^) compared to Israeli men (2,205 METs-min_*_wk^-1 ^*p *< 0.0001), or to Palestinian and Israeli women, who had similar medians (2776 METs-min_*_wk^-1^). Two thirds (65%) of the total MVPA reported by Palestinian women were derived from domestic chores compared to 36% in Israeli women and 25% among Palestinian and Israeli men. A high proportion (63%) of Palestinian men met the PA recommendations by occupation/domestic activity, compared to 39% of Palestinian women and 37% of the Israelis. No leisure time PA was reported by 42% and 39% of Palestinian and Israeli men (*p *= 0.337) and 53% and 28% of Palestinian and Israeli women (*p *< 0.0001). Palestinian women reported the lowest level of walking. Considering all domains, 26% of Palestinian women were classified as insufficiently active versus 13% of Palestinian men (*p *< 0.0001) who did not differ from the Israeli sample (14%). Middle-aged and elderly and less educated Palestinian women, and unemployed and pensioned Palestinian men were at particularly high risk of inactivity. Socio-economic indicators only partially explained the ethnic disparity.

**Conclusions:**

Substantial proportions of Palestinian women, and subgroups of Palestinian men, are insufficiently active. Culturally appropriate intervention strategies are warranted, particularly for this vulnerable population.

## Background

The growing burden of cardiovascular disease (CVD) in low-and middle-income countries has been extensively documented [1,2]. Projections that are based solely on demographic shifts suggest that the Eastern Mediterranean region will experience the largest increase in ischemic and cardiovascular mortality in the world by 2020 [2]. Within this region, urban Palestinians appear to be especially vulnerable. Compared with 20 population-based centers that participated in the World Health Organization MONICA program in the early to mid 90s, Palestinians from east Jerusalem showed the highest incidence both of total coronary heart disease (CHD) and non-fatal myocardial infarction [3].

Effective prevention and control of CVD among Palestinians requires knowledge of their CVD risk factors. A cross-sectional study conducted between 1996 and 1998 documented alarming high prevalence rates of obesity (42%), hypertriglyceridemia (35%), and low HDL-cholesterol (61%) among urban Palestinians living in the city of Ramallah, whereas the corresponding rates in a nearby rural community were 28%, 23% and 28% [4]. The reported prevalence of type 2 diabetes was 12% in both communities, while 21% had hypertension and 17% were classified as having the metabolic syndrome [4]. A much higher prevalence of the metabolic syndrome (33%) was documented in 2005 for an east Jerusalem sample by the same criteria [5].

Physical activity (PA) is an established key modifiable factor of all these conditions [6,7]. Data on PA levels of Palestinians are, however, limited. The above rural and urban communities surveyed near Ramallah may be the only available source [4]. Based on occupation title, 67% of urban men were classified into sedentary and low PA occupations (i.e., clerical, sales, and included retired/unemployed), compared with 43% of rural men. No other PA domain was reported for men. Both urban and rural women, who were asked only about their leisure-time exercise, reported high proportions of no PA (60% and 56%, respectively) [8].

These data provide only gross classifications with no dimension of quantification (i.e., intensity, frequency, and duration). Consequently, the proportion of Palestinians that achieved the recommended 'dose' of PA for the prevention of CHD and its associated risk factors remained unknown. Also, a narrow focus on one domain of PA may lead to erroneous estimation of overall physical inactivity and the incorrect identification of the population at risk. This is of particular relevance to minority women [9,10] and socioeconomically disadvantaged groups in both developing [11] and developed countries [12,13] where low participation in leisure time PA is offset by higher levels of PA at work, home, or through active transport. Furthermore, PA in adults is influenced by a diverse range of factors including age, gender, socio-economic status (i.e., education, occupation or income), and biological, psychological, cultural, social and environmental factors [14,15]. The influence of these, individually or in combination, on the patterning of Palestinians' PA has not yet been studied.

This study is intended to address these issues in order to direct public health intervention programs for urban Palestinians. Its aims are: i) to describe and quantify sources of health-enhancing PA of urban Palestinians considering all PA domains; ii) to determine the prevalence of insufficient PA according to public health recommended thresholds; and iii) to identify the socio-demographic correlates of insufficient activity. In doing so, we compared urban Palestinians from east Jerusalem to Israeli residents of the city whose CHD incidence and mortality rates were shown to be substantially lower than those of the Palestinians, [3,16]. We applied a PA instrument designed to reflect ethnic differences in epidemiological investigations [17].

## Methods

Following the Six-Day War Israel annexed east Jerusalem. Its Arab residents received the legal status of permanent residents of Israel, providing coveted access to the job market, social security and health insurance and are recorded in the national population registry. The Jerusalem Palestinian-Israeli MERC Risk Factor Study was designed to assess differences in the distribution of cardiovascular risk factors between urban Palestinians and Israelis, following the evidence for disparity in the incidence of myocardial infarction between these two populations [3].

An age-sex-stratified random sample of 2000 Palestinian residents from east Jerusalem and 2000 Israeli residents from Jerusalem aged 25-74 (comprising 200 individuals in each sex-age decile in each group) was drawn from the Israel national population registry. Participants were recruited between 2004 and 2008 by a letter of invitation and follow-up phone calls. Ineligibility criteria included inability to provide informed consent, being institutionalized, housebound, pregnant or within 3 months of giving birth and a serious health disorder (such as metastatic cancer or end-stage renal disease). Of the Israeli sample 89.6% were eligible and 29.5% could not be located. The corresponding figures for the Palestinians were 88.4% and 25.1%. The response rates from all located eligible residents were 76.7% for Palestinians and 53.7% for Israelis.

Participants (971 Palestinians and 712 Israelis) attended two separate research centers in east and west Jerusalem, for clinical measurements and a face-to-face interview using standardized methods. Interviews, physical measurements and blood and urine collection were carried out on the same day, and were scheduled by appointments restricted to the morning hours (as participants were in the fasting state) throughout the year, except during weekends and holidays. The interview included socio-demographic characteristics (e.g. age, gender, and marital status, country of birth, education, employment status, occupation type, religion and degree of religiosity) and information about health status, family history, PA, diet, smoking and alcohol intake. The interviews were conducted in Arabic and Hebrew by trained interviewers.

All participants provided signed informed consent. The study was authorized by the St Joseph Hospital and Hadassah-Hebrew University Medical Center Ethics (Helsinki) Committees.

### Physical activity assessment, measures and data treatment

The Multi-Ethnic Study of Atherosclerosis (MESA) questionnaire, which was adapted from the Cross-Cultural Activity Participation Study (CAPS), was used [9]. The questionnaire was designed to assess PA pattern of a typical week in the past month. The questionnaire asked about 28 specific activities of different effort levels (i.e., light, moderate and hard) in the domains of work including volunteer work, home (i.e., household and outdoor chores and caring for children/adults), transport (i.e., walking to destination), and leisure (i.e., walking for recreation/exercise, dancing, sports activities, and conditioning activities). For each activity participants were asked to estimate the number of days in a week and the time per day spent doing the activity.

To estimate energy expenditure (EE) in a typical week, minutes of each activity were multiplied by its frequency and by its metabolic equivalent (MET). The same MET values as in the MESA PA scoring manual were used [18]. These were obtained from a compendium of PA [19]. Following accepted cut-off points, the MET-minutes were classified into light (< 3 METs), moderate (3-6 METs), and vigorous (> 6 METs) intensity categories. Activities of at least moderate intensity are recognized as health-enhancing [20].

The proportions of domain specific adequate PA were computed using the public health recommendations [20]. A person who met the following criteria was considered adequately active: 3 or more days of vigorous activities a week for at least 20 min per day, or 5 or more days of moderate intensity activities a week for at least 30 min per day, or 5 or more days of any combination of moderate, or vigorous intensity activities a week. The cut-off for EE of combined intensities, 600 MET-minutes per week, reflects the minimum health-enhancing threshold that was used in international PA surveys that measured all domains [21-23]. The proportions of adequate PA at work and home domains were combined to reflect a "constrained domain" that is not amenable to change; intensity is largely dictated by the nature of the occupation or the chores needed to be done, and the time spent in these domains is inter-dependent; a full time homemaker can be the person's single occupation or an addition to a part-time or full-time job.

Insufficient PA was calculated by summation of the minutes/week of vigorous intensity (5 items) and of moderate intensity (9 items) and MET-minutes of moderate to vigorous PA, hereafter defined as MVPA, from all domains (14 items, see Additional file 1: Table S2a). The combined weekly threshold for insufficient PA was the inverse of the minimal recommendation for adequate PA, that is not achieving at least 150 min/week of at least moderate intensity activity during at least 5 occasions; or 60 min of vigorous intensity at least 3 occasions; or < 600 MVPA MET-minutes a week over at least 5 occasions.

Additional estimations of health-enhancing EE and insufficient PA were derived after excluding domestic indoor chores and caring activities (i.e., for children) as scant evidence exists to support protective effect of these activities for CHD [6,24]. We also determined the proportion who reported no leisure time PA (i.e., zero MET-minutes) to allow comparison with other studies, and the proportion of those who reported walking for leisure/exercise due to the public health significance of walking [25].

Since estimates were generated between 2004 and 2008 we examined for temporal change before pooling the data from all years. There were no significant yearly differences in the mean MVPA of in the physical inactivity estimates among Palestinians and Israelis.

Twenty participants (1.0%) with inadequate PA data were excluded from the analysis (18 individuals who did not complete the PA interview and 2 whose average PA time was ≥ 25 h/day and so were considered having provided an invalid report on MESA PA questions), leaving 961 Palestinians and 701 Israelis for this analysis. This sample size is sufficient to detect ethnic differences in all-domain insufficient PA as low as 6% (type I and type II errors of 5% and 20%, respectively) assuming the international mean of 18%, or 8-10% differences in gender-specific ethnic comparisons, assuming the international means of 15% and 20% for men and women, respectively [21].

The validity of the estimated MVPA MET-minutes and vigorous MET-minutes was examined against a criterion of hand grip strength measured in kg using a hydraulic hand dynamometer (Fabrication Enterprises Inc., Irvington NY 10533, USA). The Spearman's rho for MVPA in Palestinian men and women were 0.21 and 0.34 (*p *< 0.0001), respectively and for Israeli men and women the correlations were 0.16 (*p *= 0.002) and 0.20 (*p *< 0.0001), respectively. The coefficients were somewhat higher for vigorous MET-minutes among Palestinian and Israeli men (Spearman's rho were 0.26 and 0.20, *p *< 0.0001, respectively) and Israeli women (*r *= 0.21, *p *< 0.0001) but not for Palestinian women (*r *= 0.18, *p *< 0.0001). The questionnaire was not tested for its repeatability.

### Statistical methods

All analyses are gender specific and are weighted. Post-stratified weights were calculated based on the gender-by-age distributions of Palestinian and Israeli residents of Jerusalem [26]. The statistical significance of differences in MET-minutes or weekly minutes was determined by the Wilcoxon rank-sum test, appropriate for two independent samples, due to their skewed distributions. Differences in sample characteristics and proportions of insufficient PA across population sub-groups, as well as ethnic differences within socio-demographic strata, were determined using the Pearson chi-square tests. Multivariable logistic regression models were used to identify the socio-demographic variables (independent variables) that were independently associated with all-domain insufficient PA, among Palestinians and Israelis. Age was collapsed to young (25-44), middle-aged (45-64) and elderly (65+). Marital status compared married/cohabiting to not-cohabitating (i.e., single, divorced and widowers). Education, collapsed to three categories (i.e., some high school or less, high-school or technical certificate, and university degree). Work status was collapsed differently in each gender due to small numbers in some categories; men were categorized as employed, pensioners or 'other' (e.g., unemployed, homemakers and students) and women were categorized as employed, homemakers and 'others' (e.g., unemployed, pensioners). The degree of religiosity was categorized as orthodox (including ultra-orthodox), traditional and secular. Due to small number of secular Palestinian women, the last two categories were combined in women. In combined Palestinian and Israeli gender-specific models we tested for effect modification between socio-demographic and ethnic variables, using the type 3 Wald χ ^2 ^statistics. Statistical significant was determined as *p*-value ≤ 0.05. Statistical analyses were conducted using SAS version 9.2.

## Results

Sample characteristics (Table 1)

**Table 1:** Gender-specific characteristics of the Palestinian and Israeli samples

	MEN					WOMEN				
	
	Palestinian (n = 512)		Israeli (n = 370)			Palestinian (n = 449)		Israeli (n = 331)		
	**n**	**%**	**n**	**%**	***p-value***	**n**	**%**	**n**	**%**	***p-value***

**Age group**										

25-34	76	14.8	54	14.6		61	13.6	35	10.6	

35-44	102	19.9	74	20.0	0.84	97	21.6	52	15.7	0.13

45-54	111	21.7	76	20.5		94	20.9	78	23.6	

55-64	103	20.1	86	23.2		96	21.4	81	24.5	

> = 65	120	23.4	80	21.6		101	22.5	85	25.7	

Mean (SD)	51.5 (14.0)		51.4 (13.7)			51.8 (13.7)		53.3 (13.2)		

**Marital status**										

Married/defacto	480	93.7	300	81.1		307	68.7	217	65.6	

Divorce/widow	10	2.0	35	9.5	< 0.0001	107	23.9	77	23.3	0.19

Never married	22	4.3	35	9.4		33	7.4	37	11.1	

Highest education*										
< = Primary	250	48.8	66	17.9		281	62.9	42	12.7	

Some High School	54	10.6	81	21.9	< 0.0001	33	7.4	79	23.9	< 0.0001

HSC/some college	118	23.0	91	24.7		93	20.8	79	23.9	

University degree	90	17.6	131	35.5		40	8.9	131	39.6	

Work status *										

Homemaker	19	3.8	0	0.0		393	88.1	57	17.2	

Paid work	341	67.4	260	70.3	0.0023	42	9.4	177	53.5	< 0.0001

Pensioner	77	15.2	62	16.8		1	0.2	73	22.1	

Other#	69	13.6	48	13.0		10	2.2	24	7.2	

Religiosity *										

Observant	165	32.2	146	39.7		197	44.2	119	36.2	

Traditional	310	60.5	101	27.4	< 0.0001	245	54.9	82	24.9	< 0.0001

Secular	37	7.2	121	32.9		4	0.9	128	38.9	

* numbers don't add up due to missing # unemployed, disability welfare, students & volunteer

There were noticeable ethnic differences for most socio-demographic characteristics, with the exception of age. Among men, higher proportions of Palestinians were married, had primary school education or less or were homemakers compared to Israelis, and a low proportion of Palestinian men defined themselves as secular compared to Jewish men. Among women, the majority of Palestinians were homemakers with only a small proportion reporting gainful employment; a higher proportion of Palestinians had primary school education or less compared to very low proportion of Israelis, and very small proportion of Palestinian women classified themselves as "secular" compared to Jewish women.

### Energy expenditure

Table 2 presents the energy expenditure (EE) levels of health enhancing physical activity by study groups. Among men a significant excess in EE among Palestinians was noted for moderate and vigorous intensity. By contrast, among women significant differences were noted only for vigorous activities, with Palestinian women reporting significantly lower MET-minutes than Israeli women, but no difference in total MVPA. The exclusion of household indoor chores and caring activities from the calculation of MVPA resulted in lower medians in all groups, but for Palestinian women this reduction was substantial resulting in the lowest level of MVPA, approximately one-third that of Israeli women. Within the Palestinian population strong gender disparity was evident, the MVPA of men being almost double that of women (1.7 fold) mostly due to high vigorous-intensity activity of Palestinian men, which was 20-fold higher than that of Palestinian women.

**Table 2:** Energy expenditure levels (weighted) of health enhancing physical activity^§ ^by gender and ethnicity

Men							
	**Palestinian (n = 512)**			**Israeli (n = 370)**			***P***^**#**^

	**Mean**	**median**	**Q_25_; Q_75_**	**Mean**	**median**	**Q_25_; Q_75_**	

EE (MET-min_*_wk^-1^)							

Moderate	4337	3360	1260; 6375	2681	1941	960; 3525	*P < 0.0001*

Vigorous	2166	0	0; 2520	845	0	0; 560	*P < 0.0001*

Total MVPA^a^	7161	4740	1620; 10440	3638	2205	1105; 4443	*P < 0.0001*

Total MVPA^b^	6271	3360	1080; 9135	3085	1680	670; 3795	*P < 0.0001*

**Women**							

	**Palestinian (n = 449)**			**Israeli (n = 331)**			*P*^#^

	Mean	median	Q_25_; Q_75_	Mean	median	Q_25_; Q_75_	

EE (MET-min*wk-^1^)							

Moderate	3670	2657	1200; 4950	3223	2580	1440; 4290	*0.89*

Vigorous	150	0	0; 0	344	0	0; 0	*< 0.0001*

Total MVPA^a^	3820	2778	1200; 5220	3587	2775	1650;4442	*0.45*

Total MVPA^b^	1372	630	180; 1560	2425	1778	780; 3090	*P < 0.0001*

^§ ^physical activity that raises metabolic rate by at least 3METs, here denoted as MVPA # Wilcoxon rank-sum test

^a ^MVPA = moderate to vigorous physical activity at work, all 14 items in the MESA questionnaire

^b ^MVPA = moderate to vigorous physical activity excluding household indoor chores and caring for others using (12 items)

**Figure 1:**
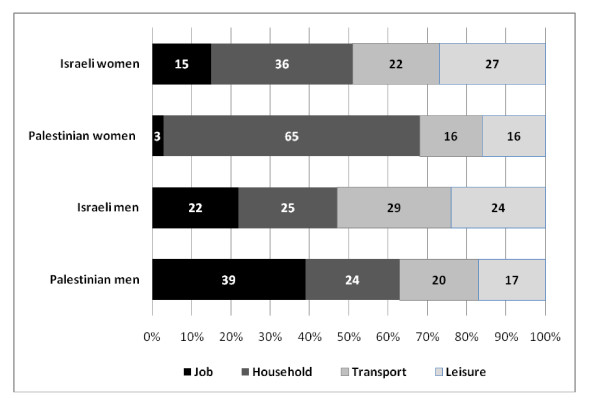
The contributions (%) of job, household, transport and leisure domains to total moderate/vigorous energy expenditure (MET-minutes_*_wk^-1^) by gender and ethnicity.

Domestic PA contributed the largest share among Palestinian women, far more than its share among Israeli women or among Palestinian and Israeli men (Figure 1). The contribution of the transport domain to total MVPA was the lowest for Palestinian women; Palestinian women reported the lowest median MET-minutes spent on travel-related walking, equivalent to 45 min a week compared to 105 min by Palestinian men and 2 h or more in the Israeli population (Additional file 1: Table S2a). Work was the main source of health-enhancing PA for Palestinian men compared to its share among Israeli men. The contribution of leisure-time MET-minutes to total MVPA was higher among Israeli men and women than among Palestinians men and women.

In the leisure domain, walking was the most prevalent mode of activity in all groups (data not shown), although a gender by ethnic interaction was evident; among men, a higher proportion of Palestinians than Israelis reported walking for exercise/recreation (53% vs 43%, *p *< 0.004) and their mean minutes of walking per week was higher (153 vs. 84 min/week, *p *= 0.002). This difference was offset by higher participation in other sports activities reported by Israelis compared to Palestinians (72 vs. 47 min per week, *p *< 0.0001), resulting in a nonsignificant difference in EE in the leisure domain. By contrast, among women, walking was less prevalent among Palestinians than Israelis (42% vs. 53%, *p *< 0.0001). The gender differences in walking levels among Palestinians were substantial: 11% more men walked for exercise than women (*p *= 0.0007) and on average men walked almost an hour (55 min) more per week than women.

### Domain-specific adequate PA, leisure-time inactivity and all-domain insufficient PA

**Figure 2:**
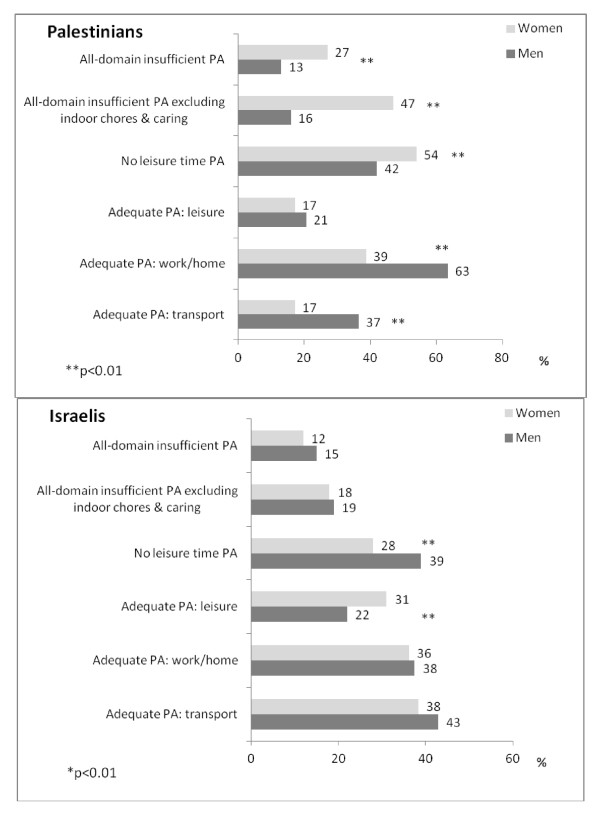
**Ethnic and gender-specific estimates (%, weighted) of domain-specific adequate physical activity (PA), leisure-time inactivity and all-domain insufficient PA with all questionnaire items or excluding moderate household chores and caring**.

There were considerable gender disparities among Palestinian for most PA indicators (Figure 2). A significantly lower proportion of Palestinian women than men achieved adequate PA at work/home or by transport. Although similar proportions of women and men achieved adequate leisure-time physical activity, inactivity at leisure (i.e., reporting no exercise) was higher among Palestinian women than men as was the prevalence of insufficient PA considering all domains or even more so without caring and domestic chores. By contrast, there were no gender differences among Israelis, except for adequate leisure- time PA and no leisure-time PA, which indicated that women were more active than men.

Gender-specific ethnic differences in adequate PA are shown in Additional file 1: Figure S2A. Among men, the proportion of Palestinians who were adequately active at work was significantly higher than Israelis, whereas in the transport domain a significant greater proportion of Israelis than Palestinians were adequately active. However, the prevalence of physical inactivity at leisure and insufficient all domain PA did not differ significantly (also after adjustment for socio-demographic differences; adjusted odds ratio (OR) = 0.76, 95% CI: 0.47-1.24).

Among women, a similar proportion of Palestinians and Israelis achieved adequate PA at work/home, but the proportions of Palestinian women classified at adequate PA at the transport and leisure domains were much lower than those reported for Israeli women. The prevalence of leisure-time inactivity and all-domain insufficient PA among Palestinian women was twice as high as that of Israelis, and almost triple the prevalence in Israeli women when indoor chores and caring were excluded (47% and 18%, *p *< 0.0001). After adjustment for socio-demographic differences, Palestinian women were twice as likely to be classified as being physically inactive by all domains as Israeli women (adjusted OR = 2.3, 95% CI: 1.3-3.8).

### Correlates of inactive lifestyle among Palestinians and Israelis

None of the socio-demographic indicators were significantly associated with all-domain insufficient activity among Palestinian men (Table 3, row %) including after adjustment for the effect of all the covariates. Among Israeli men marginal associations were noted for marital status and religiosity; married men and non-orthodox or secular men were more inactive than non-married and orthodox Israelis. We note that education was not associated with PA in either group. Among Israeli, however, the adjusted prevalence of leisure-time physical inactivity was 50% lower (*p *< 0.05) for men with a high-school certificate or university education (data not shown).

**Table 3:** Ethnic and gender-specific differences in socio-demographic correlates of all-domain insufficient PA without exclusion

	Palestinian men (N = 512)			Israeli men (N = 370)			
	
	n	% (row)	AOR^a ^(95% CI)	n	%(row)	AOR (95% CI)	***P ***^**b**^
**Age group**		*P*_(*χ*2) _= 0.187	*P*_(*χ*2) _= 0.38	*P*_(*χ*2) _= 0.33		*P*_(*χ*2 _= 0.60	

25-44	178	10.8	1.00 (ref)	128	13.7	1.00 (ref)	

≥ 45-64	214	15.8	1.25 (0.52, 2.98)	162	17.0	1.27 (0.75, 2.14)	0.37

≥ 65	120	26.7	2.05 (0.44, 9.54)	80	10.0	0.90 (0.28, 2.83)	

**Family status**		*P*_(*χ*2) _= 0.32	*P*_(*χ*2) _= 0.33	*P*_(*χ*2) _= 0.12		*P*_(*χ*2 _= 0.07	

Not cohabitating	32	6.6	1.00 (ref)	70	10.2	1.00 (ref)	

Married/defacto	480	13.6	2.35 (0.42, 13.2)	300	15.8	1.83 (0.95, 3.55)	0.32

**Education**		*P*_(*χ*2) _= 0.99	*P*_(*χ*2) _= 0.57	*P*_(*χ*2) _= 0.86		*P*_(*χ*2 _= 0.99	

< HS	304	13.2	1.00 (ref)	147	16.1	1.00 (ref)	

HSC	118	12.6	1.09 (0.45, 2.65)	91	14.2	0.99 (0.54, 1.83)	0.80

University	90	12.6	1.37 (0.47, 3.85)	131	13.7	0.98 (0.55, 1.77)	

**Work status**		*P*_(*χ*2) _= 0.09	*P*_(*χ*2) _= 0.11	*P*_(*χ*2) _= 0.92		*P*_(*χ*2 _= 0.24	

Paid job	341	10.7	1.00 (ref)	260	15.3	1.00 (ref)	

Pensioner	77	20.0	1.66 (0.41, 6.78)	62	14.3	0.59 (0.17, 2.09)	0.49

Other	88	24.7	2.45 (0.81, 7.44)	48	13.9	1.60 (0.82, 3.10)	

**Religiosity**		*P*_(*χ*2 _= 0.77	*P*_(*χ*2) _= 0.68	*P*_(*χ*2) _= 0.38		*P*_(*χ*2 _= 0.06	

Orthodox	165	14.7	1.00 (ref)	146	11.5	1.00 (ref)	

Traditional	310	12.8	0.94 (0.41, 2.16)	101	18.6	2.06 (1.11, 3.81)	0.17

Secular	37	8.9	0.64 (0.12, 3.54)	121	15.0	1.63 (0.88, 3.01)	

	**Palestinian women (N = 449)**			**Israeli women (N = 331)**			

**Age group**	*P*_(*χ*2) _< 0.0001		*P*_(*χ*2) _< 0.0001	*P*_(*χ*2) _= 0.38		*P*_(*χ*2) _= 0.043	

25-44	158	20.3	1.00 (ref)	87	13.0	1.00 (ref)	

≥ 45-64	190	33.4	2.38 (1.22, 4.62)	159	10.0	0.54 (0.29; 1.00)	< 0.0001

≥ 65	101	69.3	11.6 (3.21, 42.0)	85	15.3	1.39 (0.58, 3.35)	

**Family status**		*P*_(*χ*2) _= 0.028	*P*_(*χ*2) _= 0.82	*P*_(*χ*2) _= 0.037		*P*_(*χ*2) _= 0.014	

Not cohabitating	140	38.4	1.00 (ref)	114	8.4	1.00 (ref)	

Married/defacto	307	23.7	0.91 (0.41, 2.03)	217	14.3	2.12 (1.16, 3.88)	0.017

**Education**		*P*_(*χ*2) _= 0.026	*P*_(*χ*2) _= 0.023		*P*_(*χ*2) _= 0.11	*P*_(*χ*2) _= 0.07	

< HS	314	29.0	1.00 (ref)	121	16.4	1.00 (ref)	

HSC	93	31.3	1.30 (0.41, 2.74)	79	10.0	0.47 (0.23, 0.96)	

University	40	7.2	0.14 (0.03, 0.71)	131	10.3	0.53 (0.28, 1.01)	0.050

**Work status**		*P*_(*χ*2) _= 0.78	*P*_(*χ*2) _= 0.067		*P*_(*χ*2) _= 0.11	*P*_(*χ*2) _= 0.10	

Paid job	42	31.3	1.00 (ref)	177	11.9	1.00 (ref)	

Housewife	393	26.2	0.32 (0.11, 0.96)	57	17.8	1.66 (0.78, 3.52)	0.227

Other^c^	11	32.3	0.04 (0.01, 2.33)	97	8.5	0.57 (0.25, 1.31)	

**Religiosity**		*P*_(*χ*2) _= 0.49	*P*_(*χ*2) _= 0.46		*P*_(*χ*2) _= 0.46	*P*_(*χ*2) _= 0.022	

Orthodox	197	28.6	1.00 (ref)	119	10.9	1.00 (ref)	

Other	249	24.8	0.79 (0.43, 1.47)	210	13.0	2.11 (1.12, 4.00)	0.29

^a ^AOR = odds ratios adjusted for all covariates in the model ^b ^P = Interaction by ethnicity ^c ^= Palestinian women in "other" comprises of pensioners (n = 1), volunteers (n = 3) handicapped (n = 4) and unemployed (n = 3)

Among Palestinian women there was a strong positive association of age with insufficient activity that was not evident in Israeli women (significant ethnic interaction). An association with marital status in Palestinian women did not persist after adjustment for age, whereas married Israeli women were significantly more inactive (significant ethnic interaction). Among Palestinian women there was a strong association between working status and educational level. Thus, after adjusting for the effect of the covariates, being a Palestinian housewife was significantly associated with a lower prevalence of insufficient activity compared to women in paid jobs, and women with university degrees were substantially less likely to be insufficiently active than women with less than high-school education. Israeli women showed no significant associations with these variables, and there was a significant ethnic interactions. Israeli orthodox women were significantly less inactive than the non-orthodox, similar to the trend seen for Israeli men, but not in Palestinian men or women.

In a combined model of Palestinian men and women (data not shown) there were significant gender disparities in the associations of all-domain insufficient PA with age and education (*p *≤ 0.05 for the interactions). The prevalence of insufficient PA among middle-aged women was twice that of Palestinian men in the corresponding age group, while in the oldest age group it was almost three-fold that of men. The association between education and insufficient PA differed strongly at the highest education level, where university educated women were less inactive than men (*p *= 0.02 for the gender-by-education interaction). Palestinian women in paid jobs were more likely to be insufficiently active, whereas for men it was the reverse (*p *= 0.10 for interaction), most likely reflecting the type of work in which each gender engaged and the strong contribution of domestic chores among Palestinian housewives.

After excluding domestic indoor chores and caring activities from the calculation of insufficient PA (Additional file 1: Table S3a), the associations between all covariates and this estimate remained the same among Palestinian men but substantial changes were noted in other groups. Palestinian housewives were more likely to be inactive than Palestinian women in paid job and the associations with age and education were attenuated. In the Israeli population the association between insufficient PA and marital status were enhanced in both genders; married Israelis were more likely to be inactive than non-cohabitating and an association with work status was evident (*P *= 0.006). Further, orthodox Israelis were no longer more active than the non-orthodox or secular.

## Discussion

This study provides important information relevant to the rapid urbanization of Palestinians in the West Bank, a process that has been linked to changes in PA pattern in the non-Western world [2,27]. This research identified urban Palestinian women as being at the highest risk of physical inactivity. Accumulation of health-enhancing EE occurs primarily by domestic indoor chores and care for children, but these women had the highest proportion of leisure time inactivity and the lowest proportion of compliance with the recommended guidelines either by travel or at leisure. High educational attainment in Palestinian women was independently protective against physical inactivity. In contrast to women, Palestinian men had the highest level of health-enhancing EE of all groups mainly due to high EE at work of vigorous-intensity. Their level of compliance with public health recommendations in each domain was markedly superior to Palestinian women. We found no significant differences in levels of insufficient PA between Palestinian and Israeli men, despite differences in the pattern of PA. By contrast, there were substantial differences both in the pattern and levels of insufficient PA between Palestinian and Israeli women to the detriment of the Palestinians.

The PA pattern of Palestinian men was similar to that of urban populations in other developing countries, including China [28,29], Vietnam [30], and Nigeria [31], where the prevalence of adequate occupational and travel PA exceeded the prevalence of adequate leisure-time PA. This, however, was not the case for Palestinian women; the proportion of Palestinian women who complied with public health recommendations in the travel domain was low as their compliance in the leisure-time domain. This, plus the fact that most Palestinian women do not presently work outside their home, contributed to a greater gender disparity within the Palestinian population than those reported in urban populations in other developing countries. In Ho Chi Min City, Vietnam and Tianjin, China, women were more active than men due to their high level of travel-related PA [29,30]. The low level of leisure-time walking reported by Palestinian women is important given that walking is the most accessible form of PA. Walking was shown to reduce PA inequality within population sub-groups [32] and over time [33]. Indeed, the lack of a significant difference between Palestinian and Israeli men in adequate leisure-time PA can be attributed to walking. The reason(s) Palestinian women do not engage in daily walking for any purpose may be culture bound and should be further explored.

### Is the prevalence of insufficient PA among Palestinians high?

Caution is needed when comparing different estimates of PA prevalence because of differences between studies in questionnaires, scoring protocol and sample scope (i.e., age range, urban vs. all state). The most appropriate comparisons might be from international surveys in which all PA domains were assessed and the prevalence criteria were based on public health guidelines [21-23]. In the World Health survey of 51 mainly developing countries, the average estimates of physical inactivity (that corresponds with our definition of insufficient PA) for subjects aged 18-69 were 15% for men and 20% for women. In three Muslim countries, the United Arab Emirates (UAE), Tunisia and Pakistan, the rates of physical inactivity among men were 39.5%, 11.5% and 13.5%, respectively (compared to 12.8% for Palestinian men), whilst among women they were 59%, 18.9% and 27.6%, respectively (vs 26.0% in Palestinian women). The UAE sample was mostly urban (89%) and therefore more comparable to our study. In another international PA survey, 42.8% of men and 37.3% of women from Saudi Arabia were classified as inactive [11]. These comparisons suggest that urban Palestinians from east Jerusalem, in particular men, are more active than other Arab urban populations, but as active as African populations or the global population.

In developed countries, leisure time is generally assumed to provide the best representation of population variations in PA and therefore most surveys only assess this domain [34]. Thus, to enable a comparison to previous studies we also considered inactivity at leisure-time only. In the Palestinian population, 42% and 54% of men and women did not engage in any leisure-time PA, and 79% and 83% of men and women, respectively, were not adequately active at leisure (Figure 2). These rates were close to those reported for Israeli Arabs, where 72% and 84% of men and women, respectively, did not engage in adequate leisure-time PA [35]. Studies of immigrants to developed countries also highlighted the high prevalence of leisure time inactivity among Arab populations. In Australia, immigrants from the Middle East were 2.6 times more likely not to engage in any exercise, including walking, compared to Australian-born men and women [36]. In the United States, in one of the largest Middle Eastern Arab communities, the majority of participants (~80%) were classified as not engaging in any regular PA [37]. The authors stated that a greater proportion of inactivity was reported among women but gender specific estimates were not provided [37]. This may indicate that, in general, large proportions of Arab populations do not engage in leisure-time PA, but as observed here, there may be some compensation by increased activity in other domains.

### Correlates of insufficient PA and ethnic differences

Reviews of the Correlates and correlates of PA in populations are consistent in showing disparity by gender (men are more active than women), age (older people are less active than younger) and by socio-economic indicators (people of high SES are more active) [14], particularly evident for education [15]. Yet many of these associations were largely confined to the leisure time domain [15]. In this respect our study adds to a better understanding of PA influences considering all domains of PA. We found that the gender and age relationships with insufficient PA differed by ethnicity. Among Palestinian the linear association with age and the gender disparity were consistent with the evidence from the above systematic reviews. Among Israelis, however, women reported higher levels of leisure time PA, whereas the systematic evidence [14] indicates that men were more active at leisure than women. Additionally, there were no differences in the prevalence of all-domain insufficient PA among Israeli men and women, also after the exclusion of indoor domestic chores and caring for children. One possible explanation may be the nature of CAPS questionnaire which was developed specifically for women [9], and therefore prompt leisure-time activities that are more common among women, (e.g. dance, calisthenics) as well as household chores common to women such as caring, and possibly neglected leisure-time activities and chores that are more common to men. By contrast the systematic evidence that showed a gender difference in leisure-time activities was based on surveillance measures of a generic question on participation in moderate and vigorous intensity activities with no prompting. As men report more vigorous-intensity activity than women they score higher on these measures.

We found no association with education among men, which can be explained by the comprehensive PA measure. Support for this is found in studies from the United States and Australia in which inclusion of occupational and household activities eliminated SES differentials in PA among men but not among women [12,13]. Further, in some Asia-Pacific countries (i.e., Malaysia, Philippines and Nauru), education was not associated with any domains of PA, including leisure [11]. In our study, however, education mitigated the ethnic disparity in insufficient PA among women, but only for those with tertiary education, whereas at the lower levels of education ethnic disparity persisted. This is in line with several studies from the United States that found that SES indicators could not fully explain ethnic/racial disparity in leisure time physical inactivity [38-41]. This highlights the possible role of other factors, such as social, cultural or environmental, in explaining the disparities in PA between Palestinian and Israeli women, emphasized also by the striking disparity between Palestinian women and men.

Previous PA surveys of the Israeli population found that secular Israeli Arabs and Jews reported greater participation in leisure-time PA compared to religious or traditional people. The association appeared to be stronger among Arabs (adjusted OR = 1.5) than among Jews (adjusted OR = 1.16) [35]. This is partly corroborated here in a combined multivariable model of all men (data not shown) where for leisure-time PA secular men were more likely to be adequately active than the orthodox (adjusted OR = 1.5, 95% CI: 1.14-2.81), evident both in Israelis (adjusted OR = 1.8, 95% CI: 1.0-2.7) and Palestinians (adjusted OR = 1.5, 95% CI: 0.5-4.9) and for leisure-time inactivity particularly in women. We found, however, that when all domains of PA were assessed, secular and traditional Jews were more likely to be insufficiently active than orthodox Jews, who had the lowest proportion of insufficiently active individuals. This unexpected finding was mainly attributed to the high proportion (51%, data not shown) of orthodox/ultraorthodox Jews who achieved adequate PA by the travel domain alone. The low car ownership of the orthodox, as well as not traveling at all on the Sabbath, are possible explanations for the added walking among the orthodox Jews.

Public health recommendations on the benefits of PA and CHD are derived from longitudinal studies that primarily measured leisure-time types of PA, with only few studies assessing occupational and transport-related PA [24]. Extrapolating the recommendation to the household domain is therefore without empirical support and is problematic as it may overestimate PA levels. This has been acknowledged also in multidomain international studies [23]. Further, the exclusion of domestic activities changed the pattern of association between PA and some correlates. Among Palestinian women housewives were no longer more active than women in paid jobs and the direction of the association was similar to that reported by Israeli women and consistent with findings of systematic reviews. Excluding domestic chores also reversed differentials in insufficient activity by religiosity, as noted above, among Israeli men and women and enhanced the differential by marital status in Israeli men and women and modestly among Palestinian men. Our finding support a detrimental effect of marriage on an active lifestyle which was also confirmed in the most updated systematic review [14].

### Limitations

Although a strength of this study is the assessment of PA using a questionnaire designed for ethnically and culturally diverse populations (i.e., item-based, comprehensive) [42], the limitation of self-reported measures in term of accuracy are well known. The Cross-Cultural Activity Participation questionnaire (i.e., the measure used in the MESA study), was developed for minority women in the United States [9], and its validity was only tested in a small sample of postmenopausal women [43]. In our study the highest correlation with MVPA MET-minutes and hand grip strength was reported among Palestinian women. It may not have the same validity for men or for Israeli women. The inclusion of a wide range of moderate-intensity activities may increase measurement error. It has been shown that recall is better for vigorous intensity activity [44,45], as was observed here between hand grip and vigorous MET-minutes. Additionally, measures that attempt to assess PA in all domains may suffer from over reporting [46]. Thus, we cannot exclude the possibility that differential measurement errors existed between genders and ethnic groups. Further, while the sample had sufficient power to detect significant differences in insufficient PA between Palestinian and Israeli men and women, the study was underpowered to detect small differences in this estimate between small sub-groups, such as by marital status and education among Israeli men and women, respectively, and by employment status in Palestinian men. Finally, our response rates, particular among Israelis were not high. Post stratification weighting by gender and age cannot correct for other characteristics that are associated with PA, and therefore our estimate may be subject to non-response bias. For example, in comparison to the population we found that our sample over-represented Israeli women with a university education and under-represented those employed among Palestinians and Israelis of both genders.

## Conclusions

This study identified urban Palestinian women, particularly middle-aged or older and with low educational attainment, to be at the highest risk of physical inactivity. Given the accessibility of walking, increasing daily walking for any purpose is likely to reduce their levels of physical inactivity and improve their health status. Socio-economic indicators only partially explained the ethnic disparity. Thus to guide public health interventions it will be essential to understand the environmental, social, and personal barriers to participation. A randomized controlled trial among obese Arab women in Israel that demonstrated a culturally-sensitive lifestyle intervention to be effective in improving PA levels and favorably affect the metabolic syndrome [47] could serve as a model in this regard.

## Competing interests

The authors declare that they have no competing interests.

## Authors' contributions

DM, conducted the analyses, interpreted the finding and drafted the manuscript. JDK, initiated and oversaw the study, obtained funding, contributed to the analyses and undertook critical revision of the manuscript. RS, managed the project, contributed to the data presentation of results and manuscript. VA, participated in examination of the Palestinian participants. HN, supervised examination of the Palestinian participants. All authors read and approved the final manuscript.

## Pre-publication history

The pre-publication history for this paper can be accessed here:

http://www.biomedcentral.com/1471-2458/12/90/prepub

## Supplementary Material

Additional file 1 **Supplemental Materials include Table 2a, Figure 2a and Table 3a**. **Table 2a**. Reported mean and median minutes per week in moderate and vigorous intensity by domain and questionnaire item for each population group. **Figure 2a**. Gender-specific and ethnic estimates (%, weighted) of domain-specific adequate physical activity (PA), leisure-time inactivity and all-domain insufficient PA with all moderate to vigorous questionnaire items or without household chores and caring. **Table 3a**. Ethnic and gender-specific differences in socio-demographic correlates of all-domain insufficient PA with exclusion of domestic chores and caring. Click here for file

## References

[B1] Yusuf S Reddy S Ounpuu S Anand S Global burden of cardiovascular diseases: part I: general considerations, the epidemiologic transition, risk factors, and impact of urbanization Circulation 2001 104 22 2746 2753 10.1161/hc4601.099487 11723030

[B2] Yusuf S Reddy S Ounpuu S Anand S Global burden of cardiovascular diseases: part II: variations in cardiovascular disease by specific ethnic groups and geographic regions and prevention strategies Circulation 2001 104 23 2855 2864 10.1161/hc4701.099488 11733407

[B3] Kark JD Fink R Adler B Goldberger N Goldman S The incidence of coronary heart disease among Palestinians and Israelis in Jerusalem Int J Epidemiol 2006 35 2 448 457 16455758 10.1093/ije/dyl012

[B4] Abdul-Rahim HF Husseini A Bjertness E Giacaman R Gordon NH Jervell J The metabolic syndrome in the West Bank population: an urban-rural comparison Diabetes Care 2001 24 2 275 279 10.2337/diacare.24.2.275 11213878

[B5] Abu Sham'a RA Darwazah AK Kufri FH Yassin IH Torok NI MetS and cardiovascular risk factors among Palestinians of East Jerusalem East Mediterr Health J 2009 15 6 1464 1473 20218139

[B6] Berlin JA Colditz GA A meta-analysis of physical activity in the prevention of coronary heart disease Am J Epidemiol 1990 132 612 628 2144946 10.1093/oxfordjournals.aje.a115704

[B7] Pate RR Pratt M Blair SN Haskell WL Macera CA Bouchard C Buchner D Ettinger W Heath GW King AC Physical Activity and Public Health. A recommendation from the Center for Disease Control and Prevention and the American College of Sports Medicine J Am Med Assoc 1995 273 5 402 407 10.1001/jama.1995.03520290054029 7823386

[B8] Abdul-Rahim HF Holmboe-Ottesen G Stene LC Husseini A Giacaman R Jervell J Bjertness E Obesity in a rural and an urban Palestinian West Bank population Int J Obes Relat Metab Disord 2003 27 1 140 146 10.1038/sj.ijo.0802160 12532166

[B9] Ainsworth BE Irwin ML Addy CL Whitt MC Stolarczyk LM Moderate physical activity patterns of minority women: the cross-cultural activity participation study J Womens Health Gend Based Med 1999 8 6 805 813 10.1089/152460999319129 10495261

[B10] Brownson RC Eyler AA King AC Brown DR Shyu YL Sallis JF Patterns and correlates of physical activity among US women 40 years and older Am J Public Health 2000 90 2 264 270 10667189 10.2105/ajph.90.2.264 PMC1446154

[B11] Bauman EA Cuevas F Omar Z Waqanivalu T Phongsavan P Keke K Bhushan A Cross-national comparison of socioeconomic differences in the prevalence of leisure-time and occupational physical activity, and active commuting in six Asia-Pasific countries J Epidemiol Community Health 2011 65 35 4312 10.1136/jech.2008.086710 20943821

[B12] Ford ES Merritt RK Heath GW Powell KE Washburn RA Kriska A Haile G Physical activity behaviors in lower and higher socioeconomic status populations Am J Epidemiol 1991 133 12 1246 1256 2063832 10.1093/oxfordjournals.aje.a115836

[B13] Salmon J Owen N Bauman A Schmitz MK Booth M Leisure-time, occupational, and household physical activity among professional, skilled, and less-skilled workers and homemakers Prev Med 2000 30 3 191 199 10.1006/pmed.1999.0619 10684742

[B14] Trost SG Owen N Bauman AE Sallis JF Brown W Correlates of adults' participation in physical activity: review and update Med Sci Sports Exerc 2002 34 12 1996 2001 10.1097/00005768-200212000-00020 12471307

[B15] Gidlow C Johnston LH Crone D Ellis N James D A systematic review of the relationship between socio-economic position and physical activity Health Educ J 2006 2006 65 338 367

[B16] Kark JD Gordon ES Haklai Z Coronary heart disease mortality among Arab and Jewish residents of Jerusalem Lancet 2000 356 9239 1410 1411 10.1016/S0140-6736(00)02849-X 11052588

[B17] Multi-Ethnic Study of Atherosclerosis Physical activity interviewer administered http://www.mesa-nhlbi.org/publicdocs/mesaexam3forms/v3_physical_activity_d.pdf Accessed 20/11/2011

[B18] Bertoni AG Whitt-Glover MC Chung H Le KY Barr RG Mahesh M Jenny NS Burke GL Jacobs DR The association between physical activity and subclinical atherosclerosis: the multi-ethnic study of atherosclerosis Am J Epidemiol 2009 169 4 444 454 19075250 10.1093/aje/kwn350 PMC2726643

[B19] Ainsworth BE Haskell WL Whitt MC Irwin ML Swartz AM Strath SJ O'Brien WL Bassett DR Jr Schmitz KH Emplaincourt PO Compendium of physical activities: an update of activity codes and MET intensities Med Sci Sports Exerc 2000 32 9 Suppl S498 S504 10993420 10.1097/00005768-200009001-00009

[B20] Haskell WL Lee I Pate RR Powell KE Blair SN Franklin BA Macera CA Heath GW Thompson PD Bauman A Physical activity and public health: updated recommendations for adults from the American College of Sport Medicine and the American Heart Association Med Sci Sports Exerc 2007 39 9 1423 1434 17762377 10.1249/mss.0b013e3180616b27

[B21] Guthold R Ono T Strong KL Chatterji S Morabia A Worldwide variability in physical inactivity a 51-country survey Am J Prev Med 2008 34 6 486 494 10.1016/j.amepre.2008.02.013 18471584

[B22] Bauman A Bull F Chey T Craig CL Ainsworth BE Sallis JF Bowles HR Hagstromer M Sjostrom M Pratt M The international prevalence study on physical activity: results from 20 countries Int J Behav Nutr Phys Act 2009 6 1 21 10.1186/1479-5868-6-21 19335883 PMC2674408

[B23] Guthold R Louazani SA Riley LM Cowan MJ Bovet P Damasceno A Sambo BH Tesfaye F Armstrong TP Physical activity in 22 African countries. Results from the World Health Organization STEPwise approach to chronic disease risk factor surveilance Am J Prev Med 2011 41 1 52 60 10.1016/j.amepre.2011.03.008 21665063

[B24] Sattelmair J Pertman J Ding EL Kohl HW Haskell WL Lee IM Dose Response between physical activity and risk of corronary heart disease. A meta-analysis Circulation 2011 124 789 795 10.1161/CIRCULATIONAHA.110.010710 21810663 PMC3158733

[B25] Lee I-M Buchner DM The importance of walking to public health Med Sci Sports Exerc 2008 40 7S S512 S518 18562968 10.1249/MSS.0b013e31817c65d0

[B26] Central Bureau of Statistics Population, locality, by age sex and religion 2006 Jerusalem, Israel

[B27] Reddy KS Cardiovascular disease in non-Western countries N Engl J Med 2004 350 24 2438 2440 10.1056/NEJMp048024 15190135

[B28] Muntner P Gu D Wildman RP Chen J Qan W Whelton PK He J Prevalence of physical activity among Chinese adults: results from the International Collaborative Study of Cardiovascular Disease in Asia Am J Public Health 2005 95 9 1631 1636 10.2105/AJPH.2004.044743 16051938 PMC1449408

[B29] Hu G Pekkarinen H Hanninen O Yu Z Tian H Guo Z Nissinen A Physical activity during leisure and commuting in Tianjin, China Bull World Health Organ 2002 80 12 933 938 12571720 PMC2567698

[B30] Trinh OT Nguyen ND Dibley MJ Phongsavan P Bauman AE The prevalence and correlates of physical inactivity among adults in Ho Chi Minh City BMC Publ Health 2008 8 204 10.1186/1471-2458-8-204 PMC2435539 18541020

[B31] Forrest KY Bunker CH Kriska AM Ukoli FA Huston SL Markovic N Physical activity and cardiovascular risk factors in a developing population Med Sci Sports Exerc 2001 33 9 1598 1604 10.1097/00005768-200109000-00025 11528351

[B32] Siegel PZ Brackbill RM Heath GW The epidemiology of walking for exercise: implications for promoting activity among sedentary groups Am J Public Health 1995 85 5 706 710 10.2105/AJPH.85.5.706 7733433 PMC1615430

[B33] Merom D Chey T Chau J Smith B Barr M Bauman A Are messages about lifestyle walking being heard? Trends in walking for all purposes in New South Wales (NSW) Australia Prev Med 2009 48 341 344 10.1016/j.ypmed.2009.02.010 19232369

[B34] Kriska AM Caspersen CL Introduction to a collection of physical activity questionnaires Med Sci Sports Exerc 1997 29 6 S5 S9 9243481

[B35] Baron-Epel O Haviv A Garty N Tamir D Green MS Who are the sedentary people in Israel? A public health indicator Isr Med Assoc J 2005 7 11 694 699 16308990

[B36] Bennett SA Inequalities in risk factors and cardiovascular mortality among Australia's immigrants Aust-NZ J Public Health 1993 17 3 251 261 10.1111/j.1753-6405.1993.tb00145.x 8286500

[B37] Jaber LA Brown MB Hammad A Zhu Q Herman WH Lack of acculturation is a risk factor for diabetes in Arab immigrants in the US Diabetes Care 2003 26 7 2010 2014 10.2337/diacare.26.7.2010 12832304

[B38] Winkleby MA Kraemer HC Ahn DK Varady AN Ethnic and socioeconomic differences in cardiovascular disease risk factors: findings for women from the Third National Health and Nutrition Examination Survey, 1988-1994 J Am Med Assoc 1998 280 4 356 362 10.1001/jama.280.4.356 9686553

[B39] Shea S Stein AD Basch CE Lantigua R Maylahn C Strogatz DS Novick L Independent associations of educational attainment and ethnicity with behavioral risk factors for cardiovascular disease Am J Epidemiol 1991 134 6 567 582 1951262 10.1093/oxfordjournals.aje.a116130

[B40] Crespo CJ Smit E Andersen RE Carter-Pokras O Ainsworth BE Race/ethnicity, social class and their relation to physical inactivity during leisure time: results from the Third National Health and Nutrition Examination Survey, 1988-1994 Am J Prev Med 2000 18 1 46 53 10.1016/S0749-3797(99)00105-1 10808982

[B41] Marshall SJ Jones DA Ainsworth BE Reis JP Levy SS Macera CA Race/ethnicity, social class, and leisure-time physical inactivity Med Sci Sports Exerc 2007 39 1 44 51 10.1249/01.mss.0000239401.16381.37 17218883

[B42] Kriska AM Ethnic and cultural issues in assessing physical activity Res Q Exerc Sport 2000 71 2 47 53 10.1080/02701367.2000.11082786 25680013

[B43] Mahabir S Baer DJ Giffen C Clevidence BA Campbell WS Taylor PR Hartman TJ Comparison of energy expenditure estimates from 4 physical activity questionnaires with doubly labeled water estimates in postmenopausal women Am J Clin Nutr 2006 84 230 236 16825700 10.1093/ajcn/84.1.230

[B44] Baranowski T Validity and reliability of self-report measures of physical activity: an information porocesing perspective Res Q Exerc Sport 1988 59 4 314 327

[B45] Durante R Ainsworth BE The recall of physical activity: using cognitive model of the question-answering process Med Sci Sports Exerc 1996 28 10 1282 1291 10.1097/00005768-199610000-00012 8897386

[B46] Rzewnicki R Vanden Auweele Y De Bourdeaudhuij I Addressing overreporting on the International Physical Activity Questionnaire (IPAQ) telephone survey with a population sample Public Health Nutr 2003 6 3 299 305 12740079 10.1079/PHN2002427

[B47] Kalter-Leibovici O Younis-Zeidman N Atamna A Lubin F Alpert G Chetrit A Novikov I Daoud N Freedman L Lifestyle intervention in obese Arab women Arch Intern Med 2010 170 11 970 976 10.1001/archinternmed.2010.103 20548010

