# Supplementary material for: Lifestyle physical activity among urban Palestinians and Israelis: a cross-sectional comparison in the Palestinian-Israeli Jerusalem risk factor study
Source: BMC Public Health. 2012 Jan 30;12:90. doi: 10.1186/1471-2458-12-90 (PMC3311574; doi:10.1186/1471-2458-12-90)
Supplement: Additional file 1 — Supplemental Materials include Table 2a, Figure 2a and Table 3a. Table 2a. Reported mean and median minutes per week in moderate and vigorous intensity by domain and questionnaire item for each population group. Figure 2a. Gender-specific and ethnic estimates (%, weighted) of domain-specific adequate physical activity (PA), leisure-time inactivity and all-domain insufficient PA with all moderate to vigorous questionnaire items or without household chores and caring. Table 3a. Ethnic and gender-specific differences in socio-demographic correlates of all-domain insufficient PA with exclusion of domestic chores and caring. [file 1471-2458-12-90-S1.DOC]

|  | **Palestinian**  **Men** | |  | **Palestinian**  **Women** | |
| --- | --- | --- | --- | --- | --- |
|  | Mean | Median |  | Mean | Median |
| Moderate -intensity | 1309 | 1020 |  | 975 | 670 |
| Work, walking, walking and carrying | 606 | 60 |  | 54 | 0 |
| Transport, walking to places | 266 | 105 |  | 132 | 45 |
| Household, indoor chores (eg. Cleaning floors) | 96 | 0 |  | 473 | 300 |
| Household, outdoor chores (eg., mowing, swipping) | 53 | 0 |  | 43 | 0 |
| Household, caring chores (eg, carrying babies, push prams) | 120 | 0 |  | 139 | 0 |
| Leisure, walking for exercise, leisure, walking the dog | 119 | 20 |  | 112 | 0 |
| Leisure, individual activities (eg, callisthenics, yoga, tai-chi ) | 14 | 0 |  | 10 | 0 |
| Leisure, conditioning (e.g., cycling, leisurely swimming etc) | 24 | 0 |  | 10 | 0 |
| Leisure, dancing | 4 | 0 |  | 1 | 0 |
|  |  |  |  |  |  |
| Vigorous intensity | 405 | 0 |  | 22 | 0 |
| Work, heavy effort (eg, builders, farm labour, loading) | 347 | 0 |  | 0 | 0 |
| Household, vigorous yard work (eg., digging, shovelling) | 28 | 0 |  | 3 | 0 |
| Leisure, dual sport (eg., tennis, racket ball) | 13 | 0 |  | 1 | 0 |
| Leisure, group sport (eg, soccer, basketball etc) | 7 | 0 |  | 1 | 0 |
| Leisure, conditioning (eg., running, swimming laps etc) | 11 | 0 |  | 16 | 0 |
|  | **Israeli**  **Men** | |  | **Israeli**  **Women** | |
| Moderate -intensity | 800 | 549 |  | 923 | 750 |
| Work, walking, walking and carrying | 322 | 0 |  | 247 | 0 |
| Transport, walking to places | 198 | 140 |  | 199 | 120 |
| Household, indoor chores (eg. Cleaning floors) | 95 | 0 |  | 220 | 150 |
| Household, outdoor chores (eg., mowing, swipping) | 21 | 0 |  | 11 | 0 |
| Household, caring chores (eg, carrying babies, push prams) | 43 | 0 |  | 70 | 0 |
| Leisure, exercise walks | 75 | 0 |  | 104 | 30 |
| Leisure, individual activities (eg, callisthenics, yoga, tai-chi ) | 11 | 0 |  | 36 | 0 |
| Leisure, conditioning (e.g., cycling, leisurely swimming etc) | 22 | 0 |  | 24 | 0 |
| Leisure, dancing | 12 | 0 |  | 11 | 0 |
|  |  |  |  |  |  |
| Vigorous intensity | 137 | 0 |  | 52 | 0 |
| Work, heavy effort (eg, builders, farm labour, loading) | 93 | 0 |  | 25 | 0 |
| Household, vigorous yard work (eg., digging, shovelling) | 12 | 0 |  | 4 | 0 |
| Leisure, dual sport (eg., tennis, racket ball) | 11 | 0 |  | 2 | 0 |
| Leisure, group sport (eg, soccer, basketball etc) | 2 | 0 |  | 0 | 0 |
| Leisure, conditioning (eg., running, swimming laps etc) | 20 | 0 |  | 21 | 0 |

**Table 2a: Reported mean and median minutes per week in moderate and vigorous intensity by domain and questionnaire item for each population group**

**
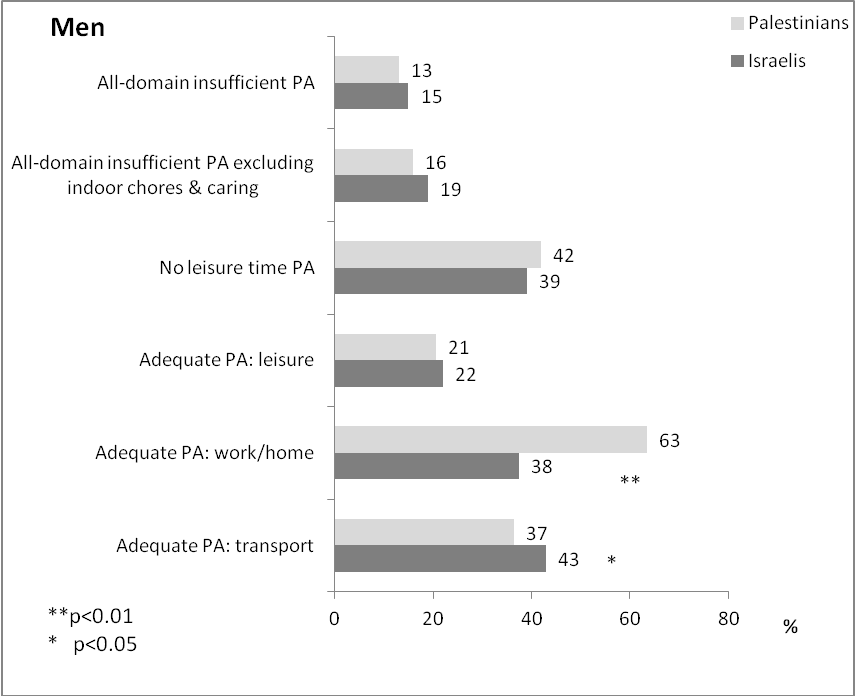
** **
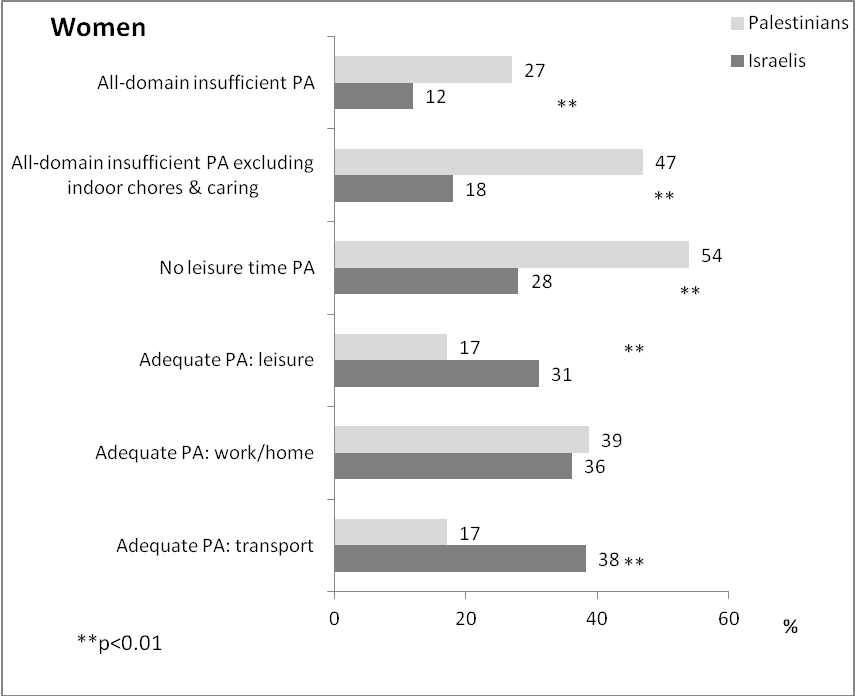
**

**Figure 2a –Gender-specific and ethnic estimates (%, weighted) of domain-specific adequate physical activity (PA), leisure-time inactivity and all-domain insufficient PA with all moderate to vigorous questionnaire items or without household chores and caring**

**Table 3a: Ethnic and gender-specific differences in socio-demographic correlates of all-domain insufficient PA with exclusion of domestic chores and caring**

|  | **Palestinian men (N=512)** | | |  | **Israeli men (N=370)** | | |  |  |
| --- | --- | --- | --- | --- | --- | --- | --- | --- | --- |
|  | n | **% (row)** | AOR^a^ (95% CI) |  | n | **%(row)** | AOR (95% CI) | *P* ^b^ |  |
|  |  |  |  |  |  |  |  |  |  |
| **Age group** |  | P_(χ2)_=.29 | P_(χ2)_=.75 |  | P_(χ2)_=.60 | | P_(χ2_=.94 |  |  |
| 25 - 44 | 178 | 14 | 1.00 (ref) |  | 128 | 19 | 1.00 (ref) |  |  |
| ≥45-64 | 214 | 19 | 1.21 (0.54, 2.70) |  | 162 | 21 | 1.07 (0.66, 1.71) | .41 |  |
| ≥65 | 120 | 28 | 1.73 (0.39, 7.66) |  | 80 | 15 | 1.15 (0.43, 3.06) |  |  |
| **Family status** |  | P_(χ2)_=.18 | P_(χ2)_=.23 |  | P_(χ2)_=.01 | | P_(χ2_=.007 |  |  |
| Not cohabitating | 32 | 7 | 1.00 (ref) |  | 70 | 11 | 1.00 (ref) |  |  |
| Married/defacto | 480 | 17 | 2.82 (0.52, 15.4) |  | 300 | 22 | 2.33 (1.26, 4.32) | .81 |  |
| **Education** |  | P_(χ2)_=.91 | P_(χ2)_=.80 |  | P_(χ2)_=.93 | | P_(χ2_=.77 |  |  |
| < HS | 304 | 16 | 1.00 (ref) |  | 147 | 19 | 1.00 (ref) |  |  |
| HSC | 118 | 17 | 1.29 (0.58, 2.88) |  | 91 | 20 | 1.22 (0.70, 2.10) | .99 |  |
| University | 90 | 14 | 1.22 (0.46, 3.25) |  | 131 | 19 | 1.13 (0.67, 1.92) |  |  |
| **Work status** |  | P_(χ2)_=.15 | P_(χ2)_=.36 |  | P_(χ2)_=.10 | | P_(χ2_=.07 |  |  |
| Paid job | 341 | 13 | 1.00 (ref) |  | 260 | 19 | 1.00 (ref) |  |  |
| Pensioner | 77 | 22 | 1.51 (0.39, 5.81) |  | 62 | 11 | 0.43 (0.13, 1.40) | .18 |  |
| Other | 88 | 27 | 2.15 (0.74, 6.24) |  | 48 | 26 | 1.66 (0.92, 3.00) |  |  |
| **Religiosity** |  | P_(χ2)_=.48 | P_(χ2)_=.58 |  | P_(χ2)_=.79 | | P_(χ2_=.99 |  |  |
| Orthodox | 165 | 20 | 1.00 (ref) |  | 146 | 21 | 1.00 (ref) |  |  |
| Traditional | 310 | 15 | 0.68 (0.32, 1.44) |  | 101 | 19 | 1.03 (0.60, 1.80) | .56 |  |
| Secular | 37 | 13 | 0.66 (0.15, 2.89) |  | 121 | 18 | 1.02 (0.60, 1.72) |  |  |
|  | **Palestinian women (N=449)** | | |  | **Israeli women (N=331)** | | |  |  |
| **Age group** | P_(χ2)_<.10 | | P_(χ2)_=.144 |  | P_(χ2)_=.38 | | P_(χ2)_=.21 |  |  |
| 25-44 | 158 | 45 | 1.00 (ref) |  | 87 | 20 | 1.00 (ref) |  |  |
| ≥45-64 | 190 | 46 | 1.06 (0.60, 1.88) |  | 159 | 14 | 0.57 (0.34; 0.94) | .11 |  |
| ≥65 | 101 | 73 | 3.50 (1.00, 12.3) |  | 85 | 17 | 1.03 (0.46, 2.28) |  |  |
| **Family status** |  | P_(χ2)_=.81 | P_(χ2)_=.85 |  | P_(χ2)_ =.010 | | P_(χ2)_=.036 |  |  |
| Not cohabitating | 140 | 46 | 1.00 (ref) |  | 114 | 12 | 1.00 (ref) |  |  |
| Married/defacto | 307 | 48 | 1.07 (0.52, 2.19) |  | 217 | 21 | 1.73 (1.03, 2.89) | .16 |  |
| **Education** |  | P_(χ2)_=.056 | P_(χ2)_=0.29 |  |  | P_(χ2)_=.54 | P_(χ2)_=.32 |  |  |
| < HS | 314 | 50 | 1.00 (ref) |  | 121 | 19 | 1.00 (ref) |  |  |
| HSC | 93 | 50 | 1.30 (0.71, 2.45) |  | 79 | 15 | 0.73 (0.39, 1.37) |  |  |
| University | 40 | 28 | 0.63 (0.24, 1.68) |  | 131 | 18 | 1.15 (0.65, 2.03) | .13 |  |
| **Work status** |  | P_(χ2)_=.09 | P_(χ2)_=.30 |  |  | P_(χ2)_=.001 | P_(χ2)_=.006 |  |  |
| Paid job | 42 | 31 | 1.00 (ref) |  | 177 | 16 | 1.00 (ref) |  |  |
| Housewife | 393 | 50 | 1.75 (0.69, 4.44) |  | 57 | 30 | 2.36 (1.26, 4.44) | .99 |  |
| Other ^c^ | 11 | 48 | 0.39 (0.27, 5.62) |  | 97 | 11 | 0.66 (0.32, 1.35) |  |  |
| **Religiosity** |  | P_(χ2)_=.29 | P_(χ2)_=.50 |  |  | P_(χ2)_=.07 | P_(χ2)_=.89 |  |  |
| Orthodox | 197 | 51 | 1.00 (ref) |  | 119 | 21 | 1.00 (ref) |  |  |
| Other | 249 | 44 | 0.84 (0.50, 1.41) |  | 210 | 15 | 1.03 (0.62, 1.72) | .84 |  |
| ^a^ AOR=odds ratios adjusted for all covariates in the model ^b^ P= Interaction by ethnicity ^c^= Palestinian women in “other” comprises of pensioners (n=1), volunteers (n=3) handicaps (n=4) and unemployed ( n=3) | | | | | | | | | |
